# Supplementary material for: Characteristics and outcomes of patients with symptomatic chronic myocardial injury in a Tanzanian emergency department: A prospective observational study
Source: PLoS One. 2024 May 1;19(5):e0296440. doi: 10.1371/journal.pone.0296440 (PMC11062551; doi:10.1371/journal.pone.0296440)
Supplement: S1 Table — (DOCX) [file pone.0296440.s001.docx]

|  | **Chronic Myocardial Injury Patients with Available Echocardiogram (N=6)** | |
| --- | --- | --- |
| **Echocardiogram Results** | **n** | **%** |
| **Overall Interpretation** |  |  |
| Global Hypokinensia | 2 | 33 |
| Pericardial Effusion | 2 | 33 |
| Multivalvular Incompetence | 1 | 17 |
| Moderate Mitral Valve Regurgitation | 1 | 17 |
| **Ejection Fraction** |  |  |
| >55% | 3 | 50 |
| 40% – 55% | 1 | 17 |
| <40% | 2 | 33 |

**S1 Table**: Echocardiogram Results of Patients with Chronic Myocardial Injury, northern Tanzania, 2020-2023
